# Insights into the biological effects of molybdenum in an insect model (*Galleria mellonella*)

**DOI:** 10.3389/fbioe.2026.1782493

**Published:** 2026-05-14

**Authors:** Brianna Hofman, Logan Florek, Sarah Atang, Amani Gillette, Babasola Fateye, Maria P. Kwesiga

**Affiliations:** 1 Cell and Molecular Biology Department, Grand Valley State University, Allendale, MI, United States; 2 Biomedical Sciences Department, Grand Valley State University, Allendale, MI, United States; 3 Biomedical Imaging, Morgridge Institute of Research, Madison, WI, United States

**Keywords:** atherosclerosis, *G. mellonella*, malpighian tubule, metabolism, molybdenum, redox balance

## Abstract

**Introduction:**

Molybdenum (Mo) is a potential biodegradable stent material that has excellent mechanical and degradation properties. Recent studies suggest that chronic exposure to Mo is associated with renal concerns. *Galleria mellonella* (*G. mellonella*) has emerged as a valid research model for assessing mechanisms and treatment for human disease. Moreover, the malpighian tubules (MT) are analogous to mammalian nephron tubules. Here, we evaluate the biological effects of Mo in MT in *G. mellonella*.

**Methods:**

Fifth instar *G. mellonella* larvae were injected with 5 μL of 0.025–200 mM doses of sodium molybdate (Mo). Survival and melanization (relative health) was assessed over a 24–72 h period. We assessed redox balance with superoxide dismutase (SOD) and malondialdehyde (MDA) colorimetric assays, tissue morphology of the MT qualitatively and quantitatively in Hematoxylin and eosin (H&E) staining of paraffin-embedded larval sections, and changes in metabolism of *ex vivo* MT using two photon fluorescence-lifetime imaging (FLIM) microscopy.

**Results:**

Mo was non-toxic at all concentrations. Colorimetric assays of MDA and SOD in hemolymph and in the MT were comparable across the treatment groups. Morphological changes were observed in the cryptonephridial complex (CNC) of the MT tubules with 10 mM Mo treatment alluding to dilation of the MT. Two photon FLIM indicated the likely presence of the metabolic species (NAD(P)H) (blue channel) with an increase in mean lifetime (Tm) and an increase of protein binding of NAD(P)H at 200 mM of Mo treatment favoring oxidative phosphorylation.

**Discussion:**

In summary, we confirmed non-toxicity of Mo in the invertebrate *G. mellonella* at the organismal and molecular level. However, morphological and metabolic changes were observed at 10 and 200 mM doses of Mo treatments suggesting possible biological alterations that could impact the performance outcomes of Mo biodegradable stents.

## 1 Introduction

Biodegradable metallic materials have become an attractive alternative to permanent vascular stents in the treatment of atherosclerotic cardiovascular disease (ACD) because of their ability to degrade overtime and facilitate a physiological vascular healing response. This unique quality reduces the complications associated with permanent implants such as in-stent restenosis and thrombus formation. (Jukema et al., 2012). The biodegradable metals currently under investigation are iron, zinc, and magnesium (Oliver et al., 2021). Research studies have shown promise with these metals. However, there are challenges that include inadequate mechanical properties, non-uniform degradation profiles, and the need for alloying which raises toxicity concerns (Guillory et al., 2022; Kwesiga et al., 2023; He et al., 2025; Sun et al., 2025).

Molybdenum (Mo) has been proposed as a candidate for biodegradable stents. Molybdenum is a transitional metal that is classified as a bio essential element. Molybdate oxyanion (MoO_4_^2−^, the main form of Mo that can be taken up by organisms) acts as a cofactor for the enzymes sulfite oxidase, xanthine oxidoreductase (XOR), and dimethylsulfoxide reductase (Schwarz, Mendel and Ribbe, 2009). Additionally, Mo has excellent mechanical properties, a uniform degradation rate, medical imaging capabilities, and minimal toxicity (Redlich et al., 2021; Schauer et al., 2021; Sikora-Jasinska et al., 2022). Although the biocompatibility of Mo for cardiovascular applications has been extensively investigated, the majority of these studies underscore the biological effects of Mo that could impact the progression of ACD (Mayers et al., 2024); specifically, Mo’s effect on XOR, a key mediator in generating oxidative stress and advancing ACD progression (Berry and Hare, 2004; Kushiyama et al., 2012; Nomura et al., 2014; Polito et al., 2021). XOR participates in purine metabolism, which involves the breakdown of hypoxanthine into xanthine and uric acid while generating superoxide (a mediator of oxidative stress) and NADH (Harrison, 2002; Berry and Hare, 2004). Additionally, the high costs and small sample sizes associated with mammalian *in vitro* and *in vivo* work can impact the validity and feasibility of Mo research studies.

Invertebrate models provide insights into the physiological and molecular impacts of treatments while reducing costs and ethical concerns. Invertebrate malpighian tubules (MT) are functionally analogous to mammalian nephron tubules processing and excreting metabolic and toxic waste within the larvae and has been used to model vertebrate kidney physiology and disease (Jung et al., 2005; Denholm, 2013; Cintra-Socolowski et al., 2016). The MT is heavily enriched with genes such as Cytochrome P450 (specifically the insect-specific Cyp6 class) and Glutathione transferase that metabolize and detoxify exogenous chemicals and oxidative species, respectively (Cintra-Socolowski et al., 2016). The MT produces the primary urine by secreting ions and organic molecules from the hemolymph into the tubule lumen and then carry excretory products (metabolic compounds like ammonia, urea, and uric acid) and toxic compounds to the hindgut or store them as minerals in the form of spherites. Malpighian tubules help maintain homeostasis by controlling fluid and ion balance of the hemolymph. Invertebrate MT rely on ATP-dependent processes to transport solutes; the high bioenergetic demands in this tissue is consistent with high mitochondrial density in the basolateral membrane of the MT principal cells (Saini, 1964; Holcombe and Weavers, 2023). It is conceivable that the MT would be susceptible to oxidative stress as a result of this high metabolic activity (Holcombe and Weavers, 2023; Liu et al., 2024; Hull, Atilano and Kinghorn, 2025). Previous work on Mo treatment in whole-body homogenates of *Drosophila* showed a dose dependent effect on oxidative stress and antioxidant markers (Perkhulyn et al., 2017). However, no study has shown how Mo may affect redox balance in specific tissues, especially in the MT.

Two-photon imaging of live tissue *in vivo* or *ex vivo* provides a unique opportunity to assess in real-time energy dysregulation in tissue. Native fluorescence, or autofluorescence (AF) is an established phenomenon which occurs when biological substrates are excited with light at a suitable wavelength (Datta et al., 2020; Georgakoudi et al., 2025). The relationship of many endogenous fluorophores with morpho-functional properties of the living systems offers a powerful resource for directly monitoring biological environments. For example, the AF proteins nicotinamide adenine dinucleotide phosphate (NAD(P)H) and flavin adenine dinucleotide (FAD) which are primarily involved in cell metabolism have been used to measure drug response in cells (Sharick et al., 2020). Quansah et al. used fluorescence lifetime imaging to show that more metabolic tissue such as the gastrointestinal tract and “tubular organs” had a longer lifetime than the less-metabolically active cuticle and demonstrated that energy imbalances occur following infection of *Galleria mellonella (G. mellonella)* (Quansah et al., 2022).

This study aims to investigate the biological effects of Mo using *G. mellonella* by evaluating (1) survival and melanization, (2) histo-morphological changes in the MT tissue, (3) the Mo impact on redox balance in the MT and hemolymph, and (4) Mo impact on relative autofluorescence and metabolism in the MT of the larvae. Together, this comprehensive assessment of the effect of Mo aims to fill gaps from previous studies and provide information about the biological effect of Mo in an *in vivo* and *ex vivo* insect model from the organismal to the molecular level.

## 2 Materials and methods

### 2.1 Molybdenum effect on survival and melanization

For the initial survival and melanization experiment, sodium molybdate (Sigma, 206370050) solutions (Na_2_MoO_4_.2H_2_O) were prepared from a stock solution of 2 M at concentrations of 0.025 mM, 0.5 mM, 10 mM, 100 mM, 200 mM. All solutions were prepared using phosphate buffered saline (PBS) solution proposed in (Djainal et al., 2020). PBS was sterilized through filtration or autoclave prior to use. Fifth instar larvae were purchased from a commercial seller (Carolina Biological) and kept at 37 °C at least 1 day prior to and during the experiments to simulate the internal body temperature of mammals. Larvae were injected with 5 µL of solutions (0, 0.025 mM, 0.5 mM, 10 mM, 100 mM or 200 mM solutions of NaMoO_4_ in PBS) for each treatment group and assessed on survival and melanization at 24, 48, and 72 h with the method for assessing melanization developed by Loh et al. (2013). The treatment groups consisted of a negative control of PBS, and a positive control of 50% dimethyl sulfoxide (DMSO) (Fisher, D128) in PBS. Three replicates were performed with 10 larvae in each treatment group in the first replicate and 7 larvae in each treatment group in the second and third replicate for a total of 168 larvae. Between injections, syringes were sterilized with a 70% ethanol solution followed by PBS solution, described above. A 1% (w/v) sodium hypochlorite was used first to sterilize the syringe between treatment groups. For injections, larvae were placed on their back and held over a 200 μL micropipette tip to expose their prolegs. The larvae were briefly washed in 70% ethanol prior to injections. Injections were performed in the last left proleg. The larvae were assessed for activity, melanization, and survival. Melanization was assessed on a scale of 0–4 with 0 consisting of complete melanization (dark brown or black color) and 4 consisting of no melanization (light tan color) representative of relative health of the larvae. Survival was assessed on a scale of 0–1 with 0 being alive and 1 being dead.

### 2.2 Histology

Larvae were fixed in 10% neutral buffered formalin (Ephredia 9400) 24 h after treatment and stored at 4 °C until they were embedded and sectioned (within 1 week) at a Core Facility (Van Andel Institute). Briefly, fixed samples were paraffin embedded and sectioned (5 um), followed by dewaxing and hydration. The tissue sections were stained using the Hematoxylin and Eosin (H&E) and imaged digitally at 20× magnification. Image analysis (lumen diameter) was performed using the Aperio ImageScope 12.4.6 software (Leica Biosystems. https://www.leicabiosystems.com/us/digital-pathology/manage/aperio-imagescope/). Lumen area was calculated using ImageJ https://imagej.net/. Lumen diameter was measured by manually circumscribing the lumen and vessel diameter. Percent of the open lumen was calculated by dividing the open (clear, non-staining) luminal area by the entire tubule area.

### 2.3 Molybdenum effect on redox balance

For assessing the impact of Mo on redox balance, larvae were treated with PBS, 10 mM and 200 mM of Mo. Hemolymph was collected after 24 h by slicing open the larvae tails and removing the hemolymph, which was placed into an anticoagulant Insect Physiological Saline (IPS) solution (150 mM sodium chloride (NaCl; Fisher BP 358–10), 5 mM potassium chloride (KCl; Sigma 7447–40), 100 mM tris(hydroxymethyl)aminomethane hydrochloride (Tris/HCl; Millipore Sigma 1082191000), 10 mM ethylenediaminetetraacetic acid (EDTA; Acros Organics (ACS) 409970025), 30 mM sodium citrate (Aldrich Chemical 6132-04-3), pH 6.9) that contains 5 ug/mL N-phenylthiourea (PTU) (Sigma P7629) (phenoloxidase inhibitor to prevent melanization)) on ice (Titball and Senior, 2020). The MT tissue was harvested into 1 mL ice cold sterile IPS containing 5 ug/mL PTU before centrifugation and homogenization. Hemolymph from 4 – 6 larvae was harvested directly into 50 μL of ice-cold IPS with 5x PTU. The samples were centrifuged and the supernatant was stored at −80 °C (protein content and Malondialdehyde assays) prior to experiments or used fresh (superoxide dismutase assay). Tissues were lysed and centrifuged according to manufacturer’s’ kit procedures. The Bradford Assay (Bradford reagent, Bio-Rad (5000205) and bovine serum albumin, Millipore Sigma (A8022)) was utilized to standardize the results for protein content. Malondialdehyde (MDA) and superoxide dismutase (SOD) within the hemolymph and MT were measured using colorimetric assays (Abcam, ab233471) and (Millipore-Sigma CS0009) respectively. The absorbance was read using a spectrophotometer (BioTek Epoch plate reader, 160203A) at absorbances 695 nm and 450 nm respectively.

### 2.4 Molybdenum effect on mean fluorescence lifetime and intensity of autofluorescence signal

For assessing metabolic impact of Mo, larvae were treated with PBS, 10 mM Na_2_MoO_4_, 200 mM Na_2_MoO_4_, 50 µM Carbonylcyanide-p- trifluoromethoxyphenylhydrazone (FCCP; Sigma Aldrich, MO C2920), dissolved in 10% DMSO in PBS, 2-Deoxyglucose 25 mM (2DG; Millipore Sigma D8375), and 4 mM Sodium Cyanide (NaCN) [Millipore Sigma, 380970]. The Na_2_MoO_4_ solution was prepared with a 2 M stock solution in PBS. All solutions used were sterilized with filtration or autoclave prior to use. The larvae were injected and incubated at 37 °C for 24 h. For the NaCN treatment group, larvae were injected and incubated for 45 min. For the FCCP treatment group, larvae were injected and incubated for 15 min. For the 2DG treatment group, larvae were injected and incubated for 60 min. Timings were chosen based on when expected peak metabolic shifts would occur. Larvae were laid on their back and their tails were cut open to allow for access to their MT. The MT were removed from only live larvae and placed in a solution of sterile IPS. Dissected MT were then placed in dishes with sterile deionized water to avoid drying out of the sample during imaging.

The samples were imaged on a custom built two-photon microscope (Bruker Fluorescence Microscopy) using a 20× air objective (FOV – 660 × 660 um) with 1x optical zoom. For two-photon excitation, an ultrafast tunable laser (Insight DS+, Spectra-Physics) was tuned to 750 nm for blue channel excitation, and 890 nm for green channel excitation. Bandpass filter cubes (Chroma) were used to isolate blue fluorescence emission from 400 to 480 nm, and green emission from 500 to 600 nm. A GaAsP photomultiplier tube (H7422, Hamamatsu) was used to detect emitted photons and time correlated single photon counting electronics (SPC, Becker and Hickl) with Prairie View software (Bruker Fluorescence Microscopy) was used for lifetime imaging. Fluorescence lifetime decay curves were acquired from 256 × 256 pixel images integrated for 60 s, with a pixel dwell time of 4.8 µs. Power at the sample was maintained at less than 9 mW for the blue channel and less than 15 mW for the green channel. An instrument response function was measured from second harmonic generation of urea crystals, using an excitation of 890 nm, and the full width at half maximum (FWHM) was ∼220 ps. A fluorescent YG bead (Polysciences Inc.,Warrington, PA) was imaged as a daily reference and the expected lifetime of 2.13 ± 0.03 ns was consistent with published values (Sharick et al., 2018). A minimum of 6 larvae were imaged per condition, with one MT sample taken from each and imaged with three representative fields of view.

Lifetime images were analyzed with SPCImage software (SPCImage, version 7.8, Becker and Hickl GmbH), a bin of 9 surrounding pixels (3 × 3) was used to increase the fluorescence counts in each decay, and a threshold was used to exclude pixels with low fluorescence signal (Sharick et al., 2020). The fluorescence lifetime decay curve was deconvolved with the instrument response function and fit to a two-component exponential decay I(t) = α1*exp (-t/τ1) + α2*exp (-t/τ2) +C, where I(t) is the fluorescence intensity at time t after the laser pulse, α represents the fractional contribution of the component, τ represents the fluorescence lifetime of each component and C accounts for background light. A two-component model is used because the most likely fluorophores in the blue and green channels, NAD(P)H and FAD respectively, exist in bound and unbound states. Mean fluorescence lifetimes (τm) were calculated using the equation τm = α1τ1+ α2τ2. When fitting lifetime decays, shift values were iterated per image to minimize the X^2^ values 1.1 ± 0.1.

Segmentation was performed on all images analyzed using CellProfiler (Version 4.2.6) to isolate the autofluorescence in the lumen wall that is representative of the MT epithelial lining and eliminate background signal from other autofluorescent tissue compartments.

### 2.5 Statistical analysis

The survival analysis was performed using the Kaplan-Meier curve followed by a *post hoc* with Bonferroni correction for multiple comparisons. Melanization (relative health), histological analysis, metabolic assessment, MDA, and SOD levels data were analyzed using GraphPad Prism 10. The data is presented as the mean ± standard deviation. Statistical significance was tested using a one-way ANOVA followed by a *post hoc* Dunnett test or Tukey’s multiple comparison test. (Differences were considered significant for a p value ≤0.05.

## 3 Results

### 3.1 Molybdenum effect on survival and melanization

**FIGURE 1:**
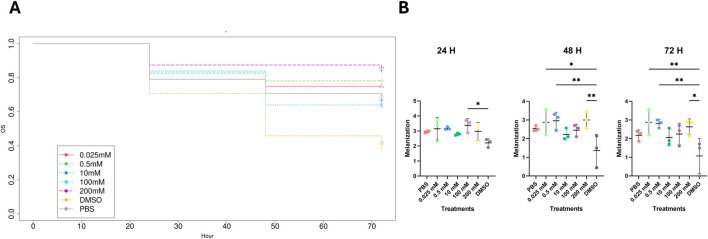
Survival analysis **(A)** and melanization (relative health) **(B)** for *G. mellonella* treated with PBS (negative control), 0.025–200 mM of Mo, and 50% DMSO (positive control) at 24, 48, and 72 h. N = 3 replicate experiments for each treatment group*P < 0.05. DMSO vs. 200 mM Mo (Chisq = 10 on 1 DF, p = 0.002); 100 mM (Chisq = 3.8 on 1 DF, p = 0.05); 10 mM (Chisq = 3.2 on 1 DF, p = 0.07); 0.5 mM Mo (Chisq = 7 on 1 DF, p = 0.008); 0.025 mM Mo (Chisq = 5.8 on 1 DF, p = 0.02). DF = degrees of freedom.

Larvae were dosed with PBS (negative control), 0.025, 0.5, 10, 100, 200 mM Mo, or DMSO (positive control) and observed for 24, 48, and 72 h. The survival and melanization (relative health of the larvae were recorded, and the experiment was repeated three times in replicates of 10, 7, and 7. Individual replicates for survival are presented in Supplementary Figure S1. There was no significant difference between Mo treatments and PBS. The larvae treated with Mo had a trend of better survival rates compared to larvae treated with DMSO from 24–72 h demonstrating the non-toxicity of Mo (Figure 1A). The Mo treatments had survival rates ranging between 60% and 85% at 72 h with similar survival rates throughout. The 10 mM Mo treatment group had the lowest survival rate of the Mo treatment groups after 24 h at 60%. Relative health was recorded at 24, 48, and 72 h on a 0–4 scale for relative health with 4 representing no melanization and 0 representing complete melanization (Figure 1B). At 24 h relative health was non-significant among treatment groups except for 100 mM which had better relative health compared to DMSO (P = 0.0189). At 48 h better relative health was observed for 0.025, 0.5, and 200 mM of Mo treatment group vs. DMSO (P = 0.0126, 0.0087, and 0.0072 respectively). Relative health for 0.025, 0.5, and 200 mM of Mo treatment groups remained consistent at 72 h (P = 0.0069, 0.0091, and 0.0191 vs. DMSO respectively) Overall, DMSO treated larvae consistently showed a trend of low relative health compared to all treatment groups.

### 3.2 Acute effects of molybdenum on tissue morphology

**FIGURE 2:**
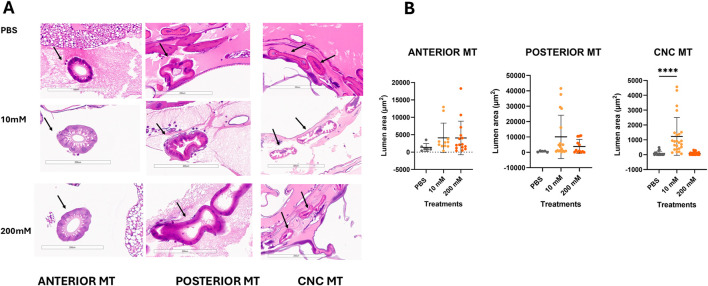
Histological analysis of malpighian tubules (MT) for *G. mellonella* treated with PBS (negative control), 10 and 200 mM of Mo, at 24 h. Qualitative **(A)** and quantitative **(B)** analysis of lumen size for posterior, anterior and (CNC)MT. Black arrows represent MT viewed within tissue sample. Scale bar 200 µm *P < 0.05, **P < 0.01, ***P < 0.001 (N of 5–35 per group).

Hematoxylin and eosin stain of longitudinal sections of larvae 24 h after treatment with PBS or Mo are shown in Figure 2. The structure of the MT from different regions of the larval body - anterior, ileac plexus (posterior) and the cryptonephridial complex (CNC) are presented in Figure 2A. The organization of the different MT in *G. mellonella* larvae is demonstrated in Supplementary Figure S2A. MTs in the anterior to mid-gut area were all patent, with uniform circular morphology and did not differ histologically. Posterior MT were all large with non significant variability in lumen area. The CNC MT appeared to be dilated in 10 mM treatment compared with the PBS control. The CNC MT in 10 and 200 mM Mo treated larvae appear to have eosinophilic content in the lumens (Supplementary Figure S2C). This may suggest fluid flux and/or other contents in their lumen. The lumen area of the inner MT of the CNC were significantly higher for 10 mM Mo compared to the PBS control (P < 0.0001) (Figure 2B). Interestingly, the MT lumen area of 200 mM Mo treatment was non significant compared to PBS treatment. The lumen area for CNC MT was found to be decreased compared to anterior and posterior MT independent of Mo treatment (Supplementary Figure S2B). Luminal patency was significantly lower in 10 mM treatment compared to PBS in anterior and increased in CNC MT (Supplementary Figure S2B IV).

### 3.3 Molybdenum effect on redox balance

**FIGURE 3:**
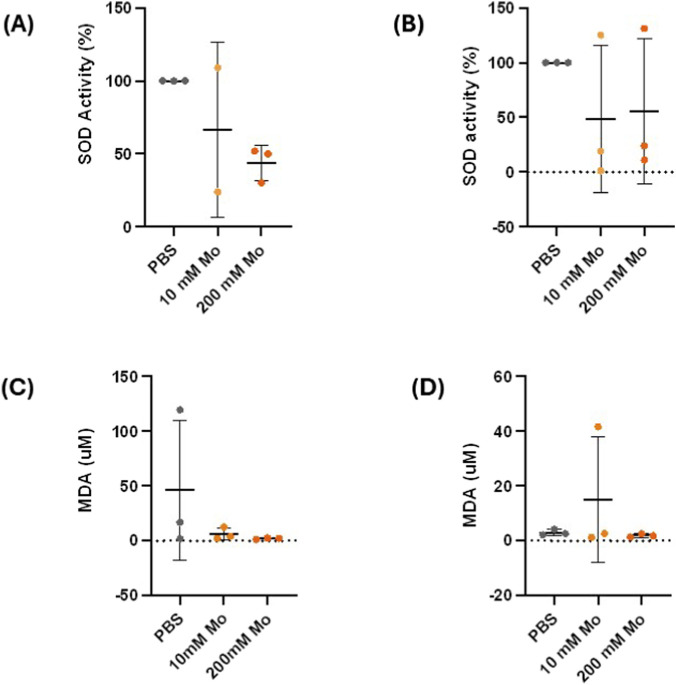
Redox balance analysis of malpighian tubules (MT) and hemolymph for G. mellonella treated with PBS (negative control), 10 and 200 mM of Mo, at 24 hours. Percentage of SOD activity to the PBS control in MT **(A)** and hemolymph **(B)**. Malondialdehyde (MDA) levels of MT **(C)** and hemolymph **(D)** for *G. mellonella* treated with PBS (negative control), 10 and 200 mM of Mo, at 24 hours. N = 3 replicate experiments for each treatment group.

Varying doses of Mo treatment showed no signs of toxicity in *G. mellonella* larvae for survival experiments. Therefore, 10 mM and 200 mM of Mo treatment were chosen to assess acute changes in redox balance with PBS as the negative control (Figure 3). Malondialdehyde (MDA), a byproduct of lipid peroxidation was used as an indicator of oxidative stress while SOD, an enzyme that scavenges superoxide was used to investigate effects of Mo on antioxidant mechanisms. Redox balance was assessed in the hemolymph and MTs. The SOD activity was reported as percentage change compared to PBS. In the MTs 200 mM consistently showed a non-significant decrease in SOD activity while variability was observed for 10 mM Mo treatment. Similarly, SOD activity showed non-significant changes in the hemolymph (Figures 3A,B). Overall Mo showed non-significant changes in MDA levels compared to PBS treated larvae for hemolymph and MT (Figures 3C,D).

### 3.4 Molybdenum effect on mean fluorescence lifetime and intensity of NAD(P)H and FAD

**FIGURE 4:**
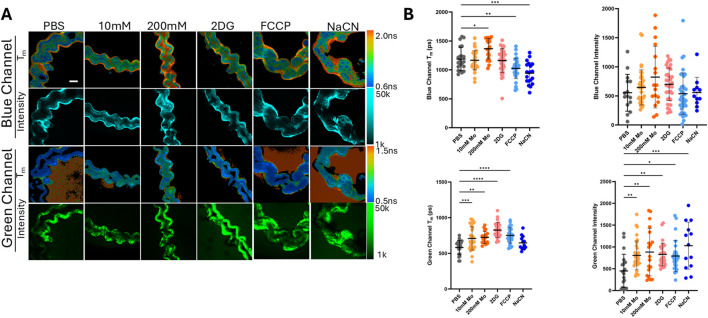
Representative images **(A)** of the mean fluorescence lifetime (Tm) and intensity of autofluorescence signal in malpighian tubules (MT) in both the blue and green spectral windows. Scale bar: 100 μm. Quantitative analysis of the MT epithelial lining **(B)** for *G. mellonella* treated with PBS (negative control), 10 and 200 mM of Mo, at 24 h 50 μM Carbonylcyanide-p- trifluoromethoxyphenylhydrazone (FCCP), 25 mM 2-Deoxyglucose (2DG), and 4 mM Sodium Cyanide (NaCN) were used as positive controls of metabolic disruption. *P < 0.05, **P < 0.01, ***p < 0.001, and ****p < 0.0001 (N of 16–37 per group).

The Mo effect on metabolism was determined by autofluorescence of signals in the two-photon blue (750ex, 440/80em) and green (890ex, 550/100em) channels which include signals from the metabolic coenzymes NAD(P)H, NADH, and FAD. Since NADPH and NADH have overlapping signals the two are reported collectively as NAD(P)H (Blacker et al., 2014). Metabolic inhibitors 2 deoxy D-glucose (2DG), which targets glycolysis, carbonyl cyanide 4-(trifluoromethoxy)phenylhydrazone (FCCP), an uncoupler of oxidative phosphorylation, and sodium cyanide (NaCN) which inhibits complex 1 of the electron transport chain were used as positive controls of metabolic disruption (Walsh et al., 2021) Phosphate buffered saline treatment was used as the negative control. 10mM and 200 mM of Mo treatments were used for determining Mo effect on metabolism. Morphologically, the epithelial lining was maintained in the wall of MT in the PBS and Mo treatment groups (Figure 4A). There was some destruction of the tissue lining observed in NaCN, however this was not consistent across all images in this treatment group. The mean fluorescent lifetime (Tm) and intensity in the blue and green spectral regions were used since they contain various autofluorescence signatures including NAD(P)H and FAD respectively. The mean fluorescent lifetime refers to the average time a fluorescing molecule remains in an excited state, while intensity is the relative amount of the molecule being excited (Datta et al., 2020). The lifetime gives information on the protein binding activity of NAD(P)H and FAD which in turn reflects changes in metabolism that favor glycolysis (decreased Tm, increase in free NAD(P)H) or oxidative phosphorylation (increased Tm, increase in NAD(P)H protein binding) (Georgakoudi et al., 2025). In the blue channel (Figure 4B), there was an increase in Tm at 200 mM of Mo treatment (P = 0.0323 vs. PBS). FCCP and NaCN treatment groups showed a decrease in Tm which is consistent with inhibited oxidative phosphorylation, when looking at NAD(P)H lifetimes (P = 0.0084 and P = 0.0010 vs. PBS). These results would indicate an increase in oxidative phosphorylation for 200 mM Mo. The fluorescence intensity in the blue channel showed a non-significant increase at 10 mM Mo and a similar trend as the Tm for 200 mM, FCCP, and NaCN. The long blue channel lifetime proportion, which is likely protein bound NAD(P)H (α2) was also found to be increased for 200 mM (Supplementary Figure S2A). In the green channel, an overlap of autofluorescence signals that likely correspond with connective tissue within the MT was observed (Datta et al., 2020). There was an increase in the Tm for 10 and 200 mM Mo (P = 0.0017and P = 0.0216 vs. PBS). These results correlated with a decrease in the protein binding (α1) of FAD (Supplementary Figure S2B). The Tm and α1 (long green channel lifetime percentage, likely bound FAD) of the green channel (FAD) decreased with both 2 DG and FCCP treatment. The fluorescent intensity in the green channel increased for all treatment groups compared to PBS.

## 4 Discussion

Molybdenum shows promise as a biodegradable metal stent with the potential to overcome the limitations of permanent metal stent implants. Studies to date have shown minimal to no toxicity of Mo for *in vitro* and *in vivo* animal models. However, little is known about the biological effects of Mo relating to ACD. The enzyme XOR incorporates Mo as a co factor, and this enzyme is found in macrophages and endothelial cells where it generates superoxide, a key regulator of redox balance in ACD (Kushiyama et al., 2012; Ives et al., 2015; He et al., 2019). The use of invertebrate models is an emerging paradigm owing to ease of use, minimal ethical concerns and larger sample sizes. *G. mellonella* in particular has been applied to infectious and toxicology studies with promising results (Astvad et al., 2017; Allegra et al., 2018; Grizanova et al., 2021). Our current work investigates Mo biological effects in *G. mellonella* in the context of mechanisms that apply to ACD. Similar to other published studies, Mo was found to be non-toxic in *G. mellonella* when assessed for survival and melanization (relative health).

The MT are analogous to kidney function in the mammalian species. Sikora et al. showed qualitative changes such as hyper cellularity and altered morphology in the glomerulus of mice kidney tissue in response to Mo treatment (Sikora-Jasinska et al., 2022). We observed that the CNC MT lumen area of the larvae treated with 10 mM Mo were dilated compared with control. This is consistent with the countercurrent mechanism of osmotic reabsorption of water from the rectum. The observation that the lumen of inner CNC MT treated with Mo 10 and 200 mM had eosinophilic staining content suggests that content of the MT was more viscous and may have other components in addition to water (Figure 2A). Acute tubular injury in mammalian kidney is characterized by lumen dilation and increased eosinophilic staining which is associated with coagulative necrosis (Gaut and Liapis, 2021). We cannot confirm the biological significance of these observations as the content of the MT vary widely from nitrogenous waste such as urates, mineral deposits (Cintra-Socolowski et al., 2016) to signaling molecules that are transported to the gut lumen (Liu et al., 2024). Additional investigations are warranted to establish a definitive evaluation.

To investigate Mo effect on redox balance we looked at changes in superoxide and MDA levels. MDA is a byproduct formed during lipid peroxidation, and it is well known as a marker for oxidative stress in various pathologies (Del Rio, Stewart and Pellegrini, 2005). In ACD it is responsible for the modification of lipoproteins that play a role in the uptake of oxLDL by macrophages, and it’s presumed to be involved in collagen crosslinking that leads to vascular stiffening observed in ACD (Viigimaa et al., 2010). Our results showed variability of MDA levels in the hemolymph and MT. The effect of Mo on redox balance has previously been investigated in the invertebrate model *Drosophila*. Molybdenum showed a dose dependent effect on oxidative stress and antioxidant markers at concentrations ranging from 0.025–10 mM (Perkhulyn et al., 2017) Low concentrations of Mo increased both antioxidant capacity and mild oxidative stress, while Mo at high concentrations caused intermediate or high intensity oxidative stress. Zhai et al. also reported that Mo is associated with a bimodal effect (antioxidative and protective at low dose and a negative effect at high doses) in sperm mice cells (Zhai et al., 2013). Interestingly Joun et al. also reported Mo’s antioxidative effects in epidemiologic studies and showed that mitochondrial SOD is upregulated in response to oxidative stress in cell-based assays (Joun et al., 2024). In our study, the oxidative stress indicator did not show a similar result which could be attributed to a difference in the oxidative stress indicator assessed, sample size, or variable effects for different types of samples. Although SOD activity showed a reduced non significant trend with 200 mM in MT which hints at mild oxidative stress.

The assessment of metabolic changes plays a crucial role in determining the efficacy or monitoring the performance of treatments. Although our survival results indicated nontoxicity of Mo, disturbances in cell metabolism are early indicators of tissue and cell damage. Furthermore, alterations in metabolism provide information on the cellular microenvironment and consequently the molecular response to treatments which in turn would affect the biological function. Multiphoton imaging is noninvasive imaging tool that facilitates real-time monitoring of endogenous fluorophores in tissue and cell samples. We assessed metabolic changes in the MT of *G. mellonella*. The results showed an increase in Tm in the blue channel (NAD(P)H) and increased protein binding which would reflect a possible increase in oxidative phosphorylation for 200 mM treatment. Oxidative phosphorylation has been found to be increased in inflammatory environments (Wculek et al., 2023) and it was also reported that this increase is crucial in lipid handling of tissue macrophages. To our knowledge this is the first time that label free imaging has been investigated in an invertebrate model with autofluorescence signatures that are representative of metabolic species. These results shed light on the molecular effects of Mo treatment that can be further explored.

Limitations of the study include using sodium molybdate instead of Mo degradation products. Sodium molybdate was used for ease of treatment through intrahemocoelic injections. Other complex compounds are formed in Mo degradation that could potentially impact survival, metabolism and redox balance in *G. mellonella*. Moreover, the use of an Mo salt solution makes it challenging to extrapolate the results to the predicted degradation rate of biodegradable metal stents (20–33 μm/year) (Oliver et al., 2021; Schauer et al., 2021; Bowen et al., 2013). The use of commercial *G*. *mellonella* could have impacted results, as their development and growth is not standardized. Standardization was done in terms of size and weight for larval developmental staging at 5th instar for survival and melanization data, but further variables could have impacted the larvae’s health and development. Additionally, hemolymph and MTs extraction had variable concentrations, and small yields for the colorimetric assays in some treatment groups with some lost in the incubation process.

Future recommendations for the study include; an increase in sample size of larvae for more accurate and consistent results, characterization of Mo biproducts post treatment using inductively coupled plasma mass spectrometry (ICP-MS) analysis, isolating hemocytes for *in vitro* studies to investigate specific molecular pathways associated with Mo treatment, and the use of an atherosclerotic research model such as pre-exposure of larvae to oxidized low density lipoprotein (oxLDL) and XOR activity assessment for direct application to ACD pathogenesis. In addition, oral treatment could provide insight into Mo’s effect on the development of the larvae. Future studies could also explore differences that occur between long-term and short-term exposure. Efficacy and safety of Mo degradation products could also be explored through inserting stent like metal implants into the larvae’s body cavity.

## 5 Conclusion

Molybdenum shows promise as a biodegradable stent candidate due to its excellent mechanical properties and non-toxicity. However, the evidence of its biological effects in the context of ACD is lacking. This study aimed to investigate the biological effects of Mo from the organismal to the molecular level by utilizing the moth larvae model *G*. *mellonella*. Survival and melanization assessment supported the non-toxicity of Mo. The increase in Tm and protein binding in the blue channel (NAD(P)H) indicated increased oxidative phosphorylation suggesting alteration of metabolism in the MT. Redox assessment showed inconsistency and variability. However, a consistent non-significant decrease in SOD activity was observed with 200 mM Mo treatment. Altogether, these results suggest biological effects of Mo that could impact the success of this biodegradable metal implant. Comprehensive investigations of the molecular mechanisms underlying ACD in the context of Mo treatment are warranted.

## Data Availability

The original contributions presented in the study are included in the article/Supplementary Material, further inquiries can be directed to the corresponding authors.
