# Supplementary material for: Insights into the biological effects of molybdenum in an insect model (Galleria mellonella)
Source: Front Bioeng Biotechnol. 2026 May 14;14:1782493. doi: 10.3389/fbioe.2026.1782493 (PMC13215999; doi:10.3389/fbioe.2026.1782493)
Supplement: Supplementary file 1 [file Supplementaryfile1.docx]

Supplementary Material

# Supplementary Figures and Tables

For more information on Supplementary Material and for details on the different file types accepted, please see [here](https://www.frontiersin.org/guidelines/author-guidelines#supplementary-material).

## Supplementary Figures


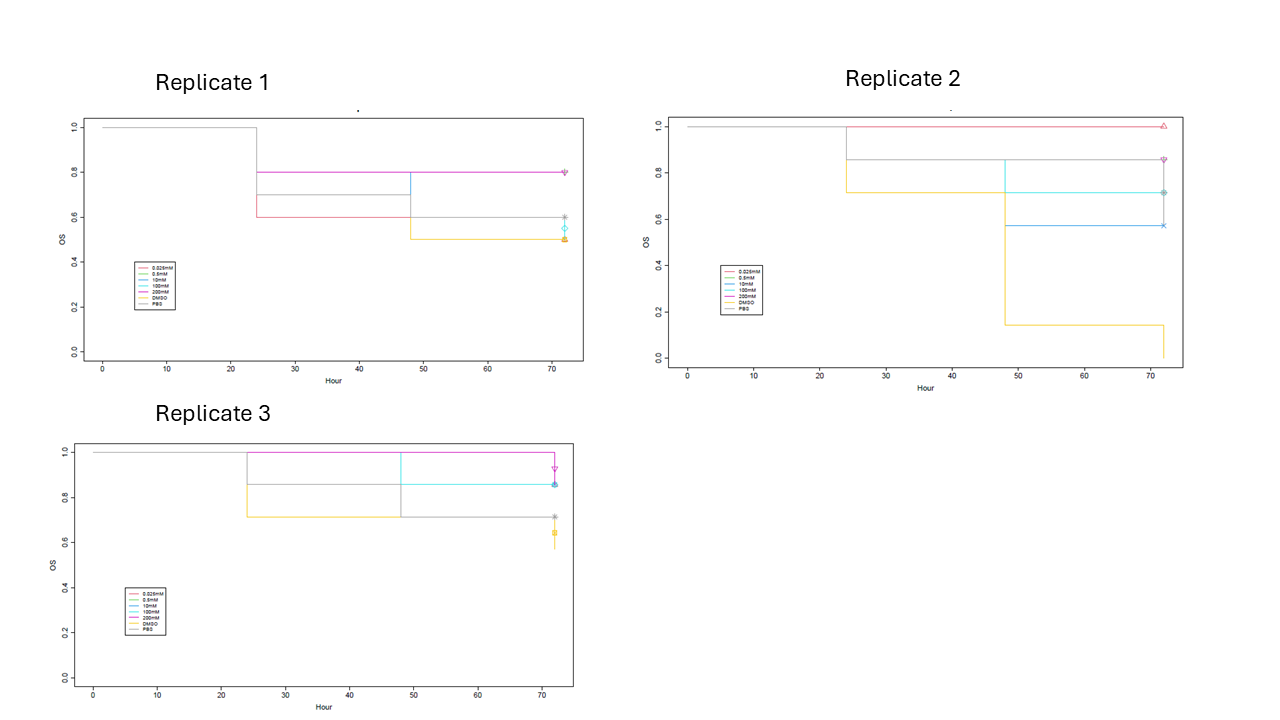


## Supplementary figure 1: Independent replicates for the survival analysis for *G. Mellonella* treated with PBS (negative control), 0.025-200 mM of Mo, and 50% DMSO (positive control) at 24, 48 and 72 hours. Repicate 1. Overall Chisq= 4.4 on 6 degrees of freedom, p= 0.6. Replicate 2. Overall Chisq= 21.5 on 6 degrees of freedom, p= 0.001. PBS vs 200mM (Chisq= 1.1 on 1DF, p= 0.3), 100mM (Chisq= 0.1 on 1 DF, p= 0.7), 10mM (p= 0.9); 0.5mM Mo (Chisq= 1.1 on 1 degrees of freedom, p= 0.3). DMSO vs PBS (Chisq= 6.7 on 1 DF, p= 0.01); 200mM Mo (Chisq= 8.6 on 1 DF, p= 0.003); 100mM Mo (Chisq= 6.2 on 1 DF, p= 0.01); 10mM Mo (Chisq= 4.3 on 1 DF, p= 0.04); 0.5mM Mo (Chisq= 8.6 on 1 DF, p= 0.003); 0.025mM Mo (Chisq= 13.9 on 1 DF, p= 2e-04). DF= degrees of freedom. Replicate 3. Overall Chisq= 3.1 on 6 degrees of freedom, p= 0.8.


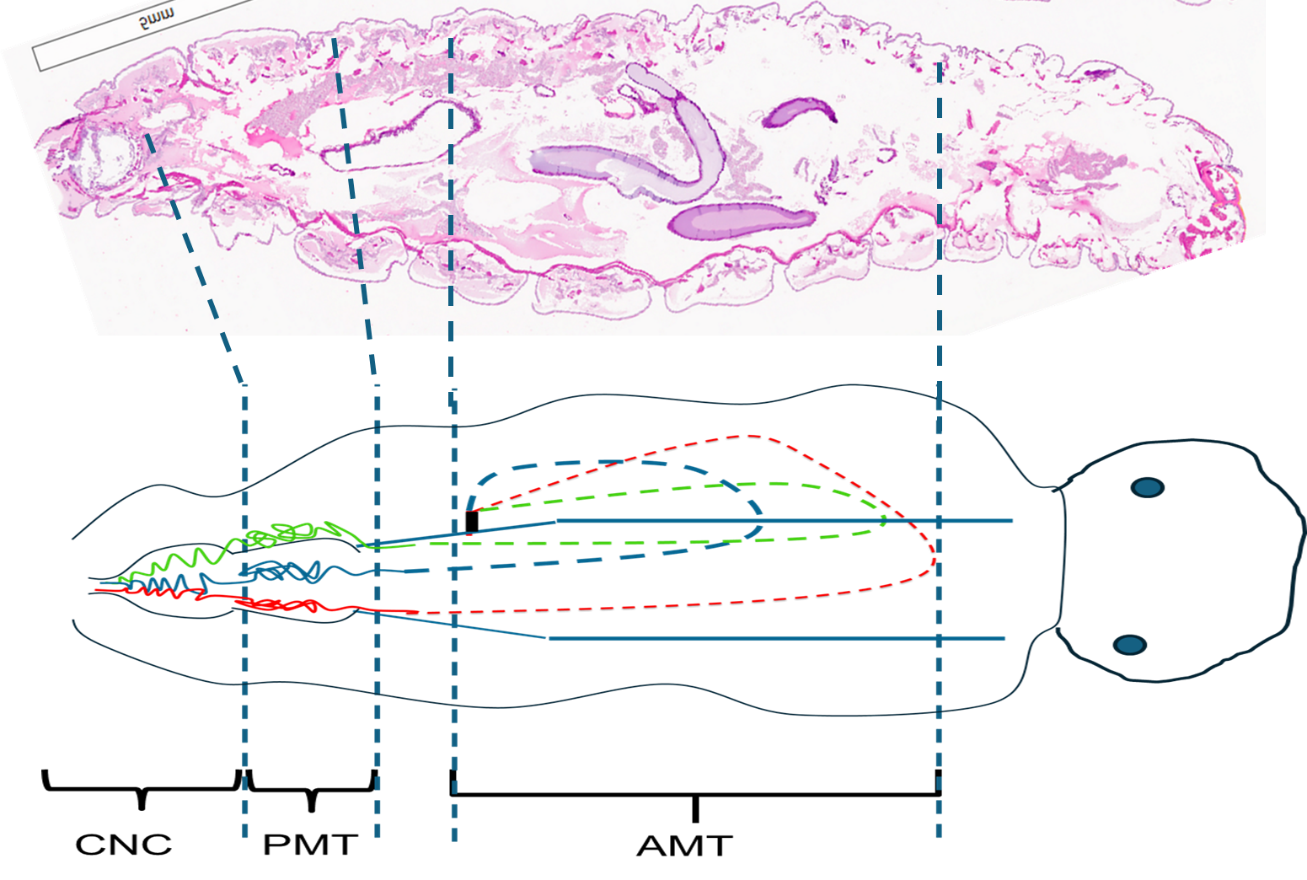


**Supplementary Figure 2A**: Organismal layout for histological analysis. In lepidopteran larvae, there are 6 Malpighian tubules that arise emerge from a pair of common ducts on either side of the colon. The tubules run anteriorly and turn around within the hemocoel (Anterior MT; AMT) towards the rear. At the posterior end, they coil up at the iliac plexus (‘posterior MT; PMT) before entering the cryptonephridial system, the coiled and smaller rectal ducts that are in direct contact with the rectum (cryptonephridial MT; CMT)(Saini, 1964; Beaven and Denholm, 2025). The juxtaposition of the rectal ducts in the CNC allows the countercurrent reabsorption of water from the rectum.


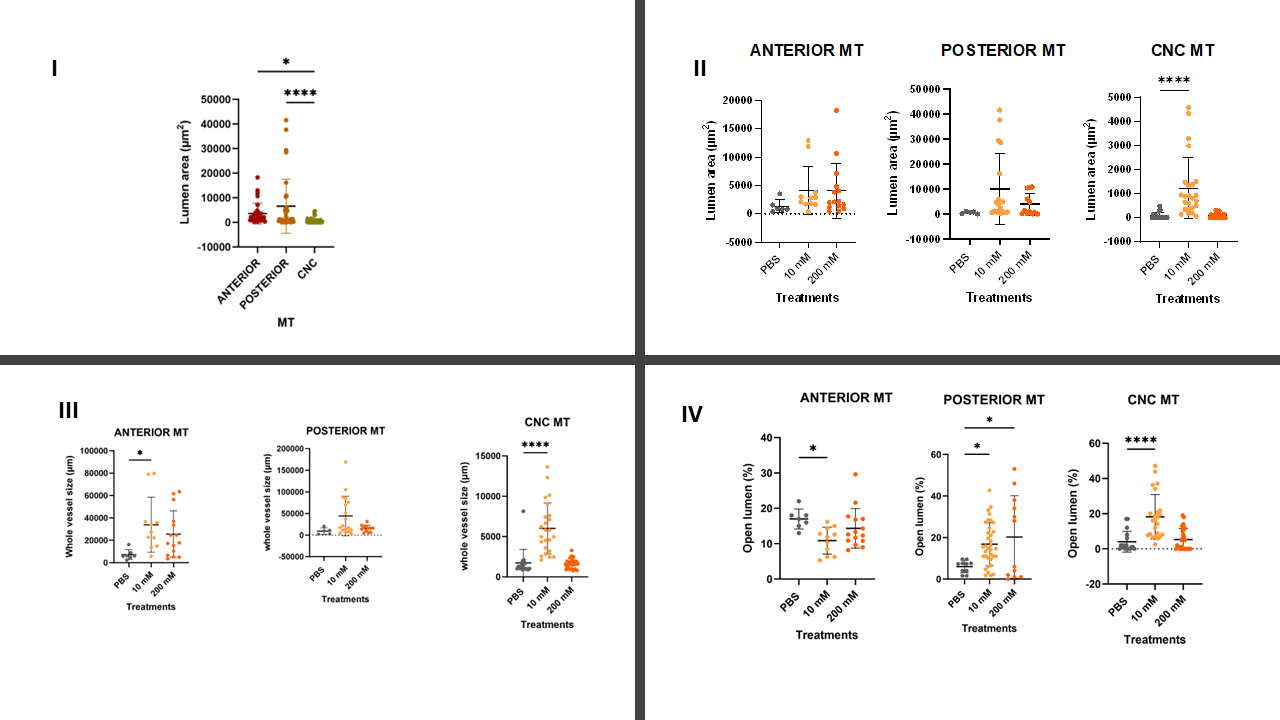


**Supplemental figure 2B.** Morphometric analysis of the MT: **I**) area of the tubule lumen diameter for anterior, posterior and CNC MT. **II)** area of the tubule lumen diameter **III)**whole vessel size, and **IV)** percentage of the lumen that is open in the 3 regions anterior, posterior and CNC Malpighian tubules for treatment groups PBS, 10 mM, and 200 mM treatments. (N of 5-35 per group). *P<0.05, **P<0.01, ***p<0.001, and ****p<0.0001


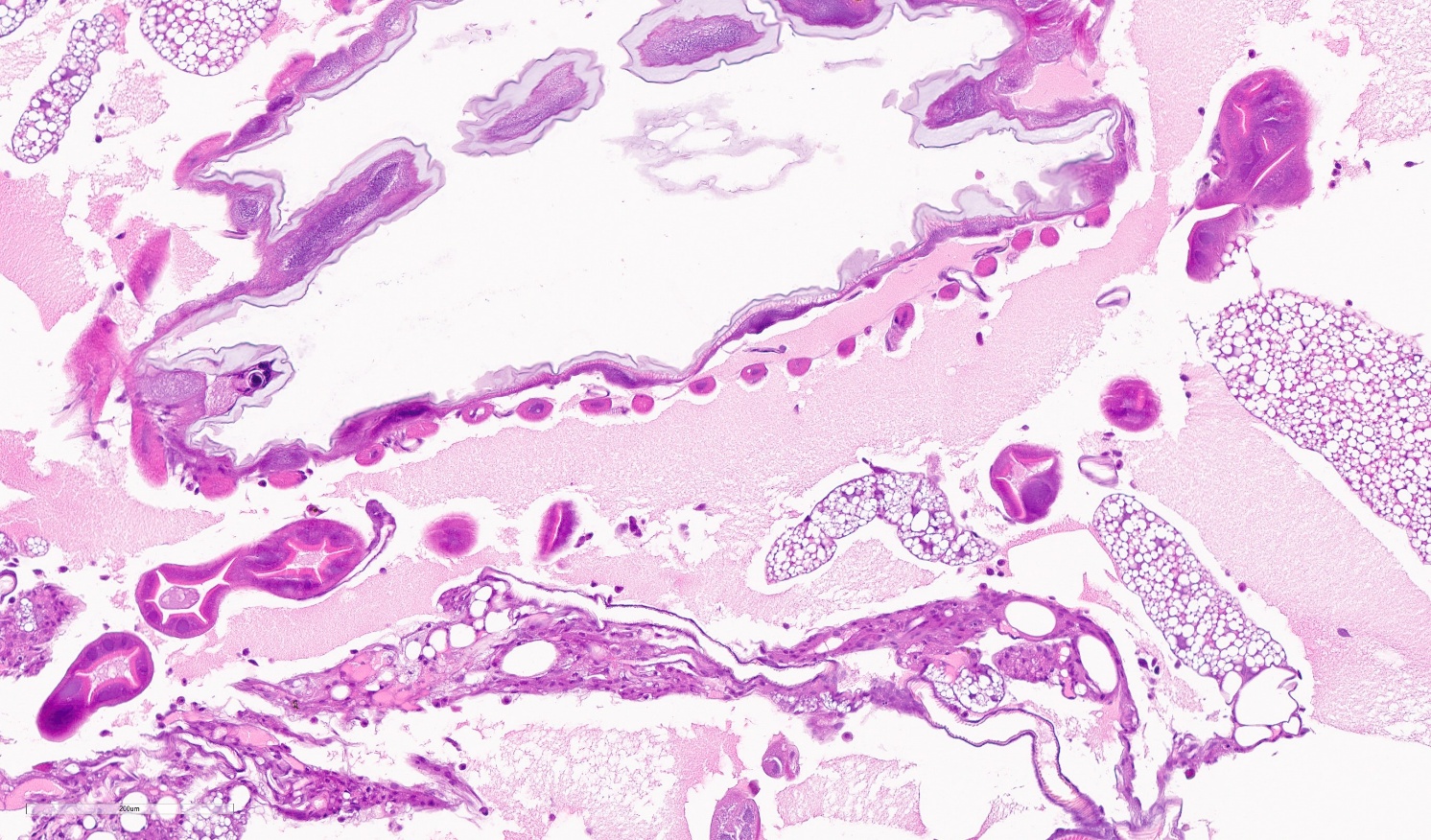


**Supplemental figure 2C.** An example of eosinophilic contents (yellow arrows) within the lumen of a posterior MT in a larva treated with 10mM Mo.


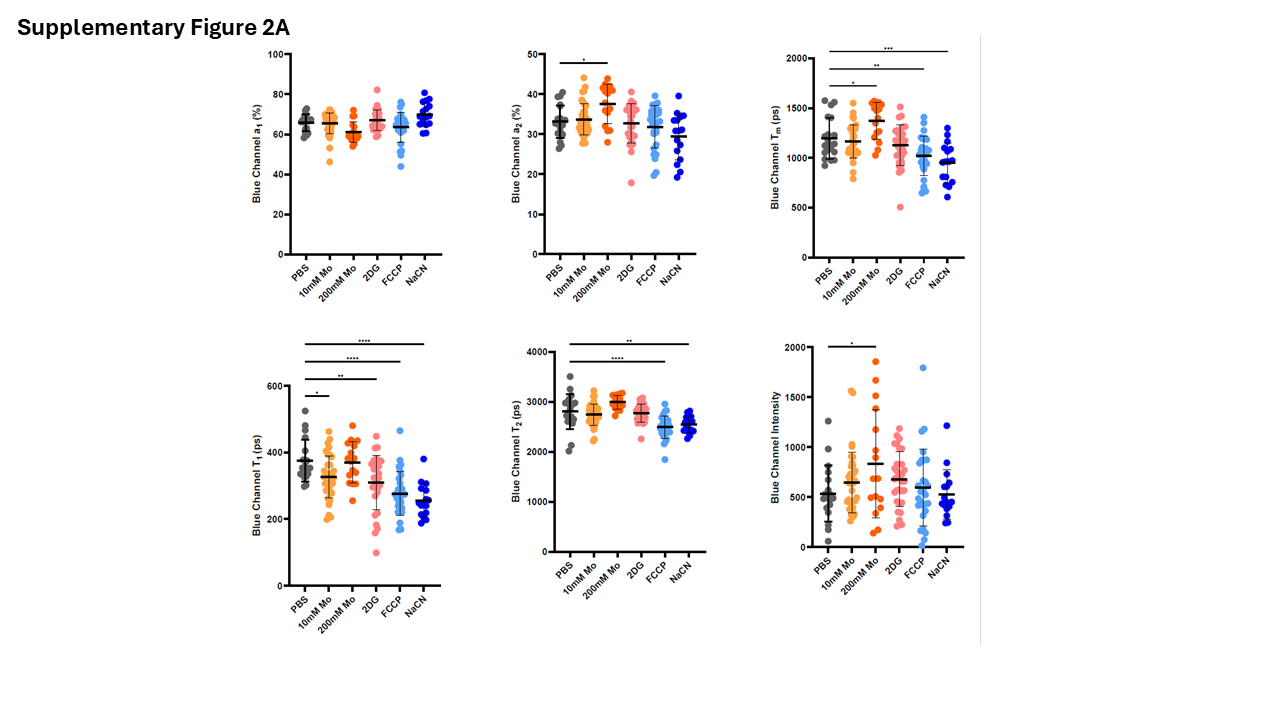


**Supplementary Figure 3A**: Quantitative analysis for *G. Mellonella* of the protein binding activity of NAD(P)H (blue channel) and the mean fluorescence lifetime (Tm) in the blue channel of Malpighian tubules (MT) treated with PBS (negative control), 10 and 200 mM of Mo, at 24 hours. 50 µM Carbonylcyanide-p- trifluoromethoxyphenylhydrazone (FCCP), 25 mM 2-Deoxyglucose (2DG), and 4 mM Sodium Cyanide(NaCN) were used as positive controls of metabolic disruption. (N of 16-37 per group). *P<0.05, **P<0.01, ***p<0.001, and ****p<0.0001


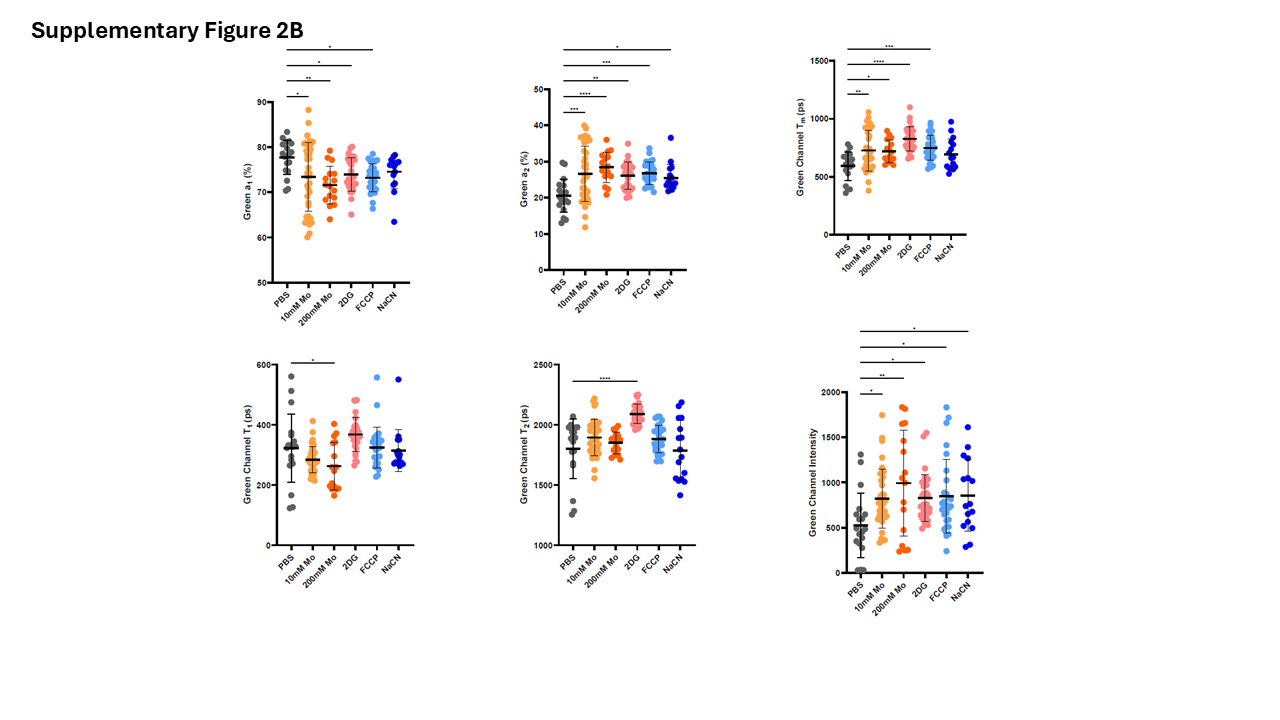


**Supplementary Figure 3B**: Quantitative analysis for *G. Mellonella and* the protein binding activity of FAD (green channel) and the mean fluorescence lifetime (Tm) in the green channel of Malpighian tubules (MT) treated with PBS (negative control), 10 and 200 mM of Mo, at 24 hours. 50 µM Carbonylcyanide-p- trifluoromethoxyphenylhydrazone (FCCP), 25 mM 2-Deoxyglucose (2DG), and 4 mM Sodium Cyanide(NaCN) were used as positive controls of metabolic disruption. (N of 16-37 per group). *P<0.05, **P<0.01, ***p<0.001, and ****p<0.0001
